# Highlight—Epigenetics Virtual Issue: New Findings Amid Old Controversies

**DOI:** 10.1093/gbe/evac019

**Published:** 2022-03-18

**Authors:** Casey McGrath

Both the term “epigenetics” and its associated field of study are relative newcomers on the scientific scene. Although the term, which suggests a process that acts “on top of” or “in addition to” conventional genetic inheritance, was first coined by Conrad Waddington in 1942, its definition has been debated ever since. Mechanisms of non-Mendelian inheritance that fall under the field of epigenetics today, including DNA methylation, histone modification, chromatin compaction, paramutation, and transgenerational inheritance, among others, were largely considered separate lines of research from the 1960s through the 1990s. In the early 2000s, these fields coalesced under the umbrella of epigenetics, although these areas of study often remain disparate. Today, epigenetics is generally defined as the study of heritable phenotypic alterations that do not involve changes in the DNA sequence, although there is still controversy over this definition and other terms in the field. Nonetheless, there has been an explosion of research into epigenetic mechanisms in recent years, aided by the emergence of next-generation technologies that have enabled deeper investigations into epigenetic patterns within species and across evolutionary timescales. The latest virtual issue from *Genome Biology and Evolution* focuses on recent articles that shed new light on the origin and evolutionary patterns of epigenetic mechanisms in diverse taxa.

The virtual issue includes three review articles that help put recent findings in epigenetics into a broader context within the field of evolutionary biology. One critical question is how conserved epigenetic mechanisms are across the eukaryotic tree of life. A team led by Agnes Weiner and Laura A. Katz used a phylogenomic pipeline to evaluate the macroevolutionary patterns of epigenetic genes among eukaryotes (Weiner et al. 2020). Their study traced a large number of epigenetic gene families back to the last eukaryotic common ancestor (LECA), while noting some differential conservation among major eukaryotic clades. This suggests that LECA may have had a genome regulated by diverse epigenetic mechanisms and that differential conservation of genes across lineages and functional categories gave rise to the punctate pattern observed today. In another review article, a collaboration between Sophie Breton, Fabrizio Ghiselli, and Liliana Milani sought to assess the influence of mitochondrial epigenetic and genetic mechanisms on animal plasticity and adaptation (Breton et al. 2021). Their literature review discusses the ways in which mitochondrial DNA methylation and mitochondria-derived noncoding RNAs and micropeptides may enable animals to rapidly respond to changing environments, which may have global implications in light of global warming and climate change. The third review by Hodson and Ross (2021) highlights evolutionary perspectives on germline-restricted chromosomes in three families of flies, each thought to have evolved this feature independently, though perhaps with elements of a shared toolkit. Notably, these germline-restricted chromosomes exhibit unusual patterns of heterochromatization and epigenetic modifications in all three fly families. According to the authors, these epigenetic features are important for a number of key aspects of these chromosomes, including their activity, elimination from somatic cells, and transmission and parent-of-origin effects. 

Two articles in the virtual issue focus on DNA methylation as a potential epigenetic mechanism to influence gene expression in bees and plants. Marshall et al. (2020) assessed the potential role of allele-specific DNA methylation on allele-specific expression in reproductive and sterile bumblebee workers. Unlike in mammals, where DNA methylation is often associated with allele-specific gene expression, the authors found no significant overlap between genes showing allele-specific expression and those with allele-specific methylation, indicating that other mechanisms are driving these gene expression patterns in bumblebees. Another study led by Brandon Gaut studied methylated CHH sequence motifs (where H indicates the nucleotides C, T, or A) in eight grass species (Martin et al. 2021). Their results recapitulated earlier findings showing that CHH methylation is associated with the nearby presence of transposable elements (TEs). However, they also found that methylated CHH islands were associated with gene body methylation and, to a lesser extent, gene length and gene expression, leaving the origin and function of this type of methylation unclear.

The role of TEs in epigenetic regulation is also discussed in a study led by Christoph Grunau and Christoph Grevelding on the human parasite *Schistosoma mansoni* (Stitz et al. 2021), a flatworm with a ZW sex chromosome system (in which males have two Z chromosomes and females have one Z and one W chromosome). Their study involved a reanalysis of repetitive W elements, which were originally thought to be found only on the W chromosome. However, the authors found that these mobile elements were not restricted to the W chromosome and that they exhibited stage-, sex-, pairing-, gonad-, and strain-specific transcription. As explained in the Highlight feature on this article (McGrath 2021a), the authors hypothesize that W elements “not only influence the biology of *S. mansoni*, but they might represent one of the sources of heritable variability, thus shaping the evolution of the family Schistosomatidae.” In another study of W chromosome repeats, Stephen H. Montgomery and coauthors describe the W chromosome in the butterfly *Dryas iulia*, which exhibits female-specific gene expression and chromatin accessibility and is characterized by highly repetitive DNA content (Lewis et al. 2021). Their findings suggest that the evolution of the W chromosome in this species may have been heavily driven by repetitive elements and that these elements may have originated from a B chromosome, a nonessential chromosome with variable presence within populations.

Two studies in the issue highlight the role of epigenetics in human evolution and adaptation. Childebayeva et al. (2021) sought to understand the epigenetic mechanisms by which exposure to high altitudes during early development influences high-altitude adaptations. By studying members of the Peruvian Quechua, who live at high altitudes in the Andes, the authors identified specific positions and regions of DNA in which methylation was associated with either lifelong or early altitude exposure, some of which were associated with genes previously linked to high-altitude adaptation. As discussed in the Highlight feature on this article (McGrath 2021b), the study supports the idea that early developmental exposures can have persistent impacts on DNA methylation patterns in humans. In their study, John A. Capra and coauthors used a machine learning method to impute gene regulation patterns in DNA samples from ancient Eurasians (Colbran et al. 2021). They found differential regulation of genes involved in metabolism and the immune system among ancient populations with different diets and lifestyles. According to the authors, this indicates that “changes in the regulation of genes in those systems contributed to recent human adaptations.”

Finally, the virtual issue includes three studies that provide further insight into diverse epigenetic mechanisms including microRNA (miRNA)-based regulation, transgenerational inheritance, and heterochromatic structures. A study by Fei Li and colleagues annotated the miRNA repertoires of 152 arthropod species (Ma et al. 2021). They found miRNA family expansions and contractions associated with evolutionary events such as morphological reduction and the origin of complete metamorphosis. In an experimental evolution study led by Michael K. Skinner and Mark Dybdahl, researchers raised three generations of clonally reproducing snails adapted to river currents in the lab without current (Smithson et al. 2020). According to the authors, their findings support the hypothesis that “adaptive shell shape variation is at least in part determined by transgenerational plasticity, and that DNA methylation provides a potential mechanism for stability of shell shape across one generation.” Finally, a Letter from Tanabe et al. (2021) describes the evolution of an unusual epigenetic adaptation in owl monkeys: accompanying their transition to a nocturnal lifestyle, these monkeys evolved a unique heterochromatin structure in the rod cells of the eye that serves as a lens to direct more light to the photoreceptor.

Together, these articles provide important context and new insights into the epigenetic mechanisms that influence phenotypic diversity across eukaryotes. By compiling them into this virtual issue of *Genome Biology and Evolution*, we hope not only to highlight recent findings in epigenetics but also to underscore open questions in the field that require additional exploration.
